# Spatial patterns of influenza A virus spread across compartments in commercial swine farms in the United States

**DOI:** 10.1080/22221751.2024.2400530

**Published:** 2024-09-02

**Authors:** Bijaya Hatuwal, Varun Goel, Thomas J. Deliberto, Jim Lowe, Michael Emch, Richard J. Webby, Xiu-Feng Wan

**Affiliations:** a Center for Influenza and Emerging Diseases, University of Missouri, Columbia, MO, USA; b Department of Electrical Engineering & Computer Science, College of Engineering, University of Missouri, Columbia, MO, USA; c Bond Life Sciences Center, University of Missouri, Columbia, MO, USA; d Department of Geography, University of South Carolina, Columbia, SC, USA; e Carolina Population Center, University of North Carolina Chapel Hill, Chapel Hill, NC, USA; f US Department of Agriculture Animal and Plant Health Inspection Service, Fort Collins, CO, USA; g Department of Veterinary Clinical Medicine, University of Illinois at Urbana-Champaign, Urbana, IL, USA; h Department of Epidemiology, University of North Carolina School, Chapel Hill, NC, USA; i Department of Geography and Environment, University of North Carolina, Chapel Hill, NC, USA; j Department of Infectious Diseases, St. Jude Children’s Research Hospital, Memphis, TN, USA; k Department of Molecular Microbiology and Immunology, School of Medicine, University of Missouri, Columbia, MO, USA

**Keywords:** Influenza A virus, swine influenza virus, transmission, commercial swine farm, farm system, migration rate, phylogeographic analyses, genetic dispersal

## Abstract

Multiple genetic variants of H1 and H3 influenza A viruses (IAVs) circulate concurrently in US swine farms. Understanding the spatial transmission patterns of IAVs among these farms is crucial for developing effective control strategies and mitigating the emergence of novel IAVs. In this study, we analysed 1909 IAV genomic sequences from 785 US swine farms, representing 33 farming systems across 12 states, primarily in the Midwest from 2004 to 2023. Bayesian phylogeographic analyses were performed to identify the dispersal patterns of both H1 and H3 virus genetic lineages and to elucidate their spatial migration patterns within and between different systems. Our results showed that both intra-system and inter-system migrations occurred between the swine farms, with intra-system migrations being more frequent. However, migration rates for H1 and H3 IAVs were similar between intra-system and inter-system migration events. Spatial migration patterns aligned with expected pig movement across different compartments of swine farming systems. Sow-Farms were identified as key sources of viruses, with bi-directional migration observed between these farms and other parts of the system, including Wean-to-Finish and Gilt-Development-Units. High intra-system migration was detected across farms in the same region, while spread to geographically distant intra- and inter-system farms was less frequent. These findings suggest that prioritizing resources towards systems frequently confronting influenza problems and targeting pivotal source farms, such as sow farms, could be an effective strategy for controlling influenza in US commercial swine operations.

## Introduction

Influenza A virus (IAV), a negative stranded and segmented RNA virus, belongs to the *Orthomyxoviridae* family and is newly classified as *Alphainfluenza* virus (influenza A virus, IAV), along with *Betainfluenza virus* (influenza B virus, IBV), *Gammainfluenza* virus (influenza C virus, ICV), and *Deltainfluenza* virus (influenza D virus, IDV). IAV has eight gene segments, encoding at least 10 proteins, including two surface glycoproteins haemagglutinin (HA) and neuraminidase (NA), three polymerases (PB2, PB1, and PA), nucleoprotein (NP), matrix proteins (M1 and M2), and non-structural proteins (NS1 and NS2). Based on antigenic subtypes of HA and NA, a total of 18 subtypes of HA and 11 subtypes of NA have been reported [1–4]. Swine, as one of the IAV natural hosts, plays a crucial role in the ecology of IAVs, and can be infected by IAVs from both avian and humans, acting as a mixing vessel for the creation of novel influenza viruses.

Since the mid-1990s in the US, multiple genetic variants of subtypes H1 and H3 IAVs, which can be categorized as classical swine lineage H1, human seasonal lineage H1, and human seasonal H3, have been co-circulating in commercial swine farms [5–7]. These enzootic swine H1 and H3 IAVs have experienced rapid evolution over the past decades, leading to the emergence of numerous genetic variants, comprising at least 22 clades and subclades of H1 and 24 clades and subclades H3 [8]. In addition, there have been sporadic spill-overs of other IAV subtypes, such as H4N6 [9–11] and H2N3 [12] from avian species, and H1 and H3 IAVs from humans [13, 14], but these spill-overs only led to limited spread.

The US is one of the largest pork producers and consumers globally, contributing approximately 20% of the total commercial production [15]. US swine farming started in the 1500s when European settlers brought pigs with them as the source of food [16]. The practices surrounding domesticated swine farming have undergone significant transformations since their initial introduction, evolving from free-range pasture grazing to various methods such as free-farmed, backyard, natural, stall-based farming, and eventually transitioning into large-scale commercial production. The industrialization of swine production began in the 1900s [17] and is primarily concentrated in the Midwestern states including Iowa, Minnesota, Illinois, Oklahoma, Indiana, Nebraska, Missouri, and Ohio and southern states including North Carolina. Swine farming operations vary widely in scale, from small family-owned farms to extensive corporate enterprises, with all sizes adhering to protocols mandated by state and federal governmental agencies, as determined by the number of pigs raised.

A typical commercial swine industry consists of a hierarchical structure comprising systems, sub-systems, and farms/sites. A system is a company that operates and manages specialized intensive farms across multiple locations. Within these systems, there may be sub-systems, which are managerial divisions created to facilitate the operational efficiency and management. Each farm within the system specializes in a particular farm type and holds pigs of specific age groups and weights. In a contemporary intensive swine production system, these farms can commonly be divided into different phases such as Gilt-Development-Unit (GDU), Farrow-to-Finish, Farrow-to-Wean (or Sow-Farm), Nursery, Wean-to-Finish, Finisher, Nursery-to-Grower, and/or Grower-to-Finish [18]. Thus, a system typically consists of multiple farms. Farms within the same system can be located within a state or spread across multiple states. Farms specializing in different swine production stages often require transporting pigs, and the movement typically occurs within the farms in the same system (intra-system) but sometimes are transported to farms in a different system (inter-system). Compared to farms where the actual biological interactions and disease transmission occur, the sub-systems primarily serve administrative functions and thus can indirectly influence disease transmission through management practices, biosecurity measures, and the movement of animals and staff within and between sub-systems. Overall, these intensive farming systems form a unique ecological environment that facilitates the transmission and evolution of infectious pathogens, including IAVs [19, 20].

In these commercial swine production systems, IAV may infect swine in any production phase and circulate throughout the year, albeit most prominently from winter through summer. High population densities on these farms inherently increase susceptibility to virus transmission and enzootic outbreaks [21]. Active surveillance in coordinated swine production systems has shown that swine IAVs can spread both within and across swine farming systems [22]. The spatial dispersal of swine IAVs can facilitate virus exposure in different pig populations, leading to prolonged circulation of certain IAV lineages and increasing the likelihood of genetic evolution [23–26]. For example, the distribution of genetic variants has been associated with pig movements, particularly from the Midwestern to southern states, such as North Carolina [27]. Network analysis of pig transportation within multiple sites of a corporate system in Iowa revealed hierarchical pig movement structures, with specific production sites playing more central roles and displaying heterogenous contact levels between farms [28]. However, it is not fully understood how IAVs disperse within and across swine farming systems, particularly across different compartments and geographic regions. Understanding these spatial dispersal patterns is crucial for identifying the factors, particularly those related to individual farming types within these compartments, that contribute to the spatial dynamics of specific genetic variants both within individual swine farming systems and across different systems.

In this study, we have curated 1909 genomic sequences of IAVs obtained from 785 individual swine farms representing 33 commercial farming systems in the US. Our dataset spans data collected from 12 states, primarily in the Midwest from 2004 to 2023. We investigated the spatial dynamics of these contemporary H1 and H3 IAV genetic clades within and across different swine farming systems. Our findings improve our understanding of the natural history of IAVs in the US swine populations and help develop effective strategies to control influenza outbreaks and mitigate the emergence of novel influenza variants in swine populations.

## Materials and methods

### Data collection from archived diagnostic samples

**Figure 1.**
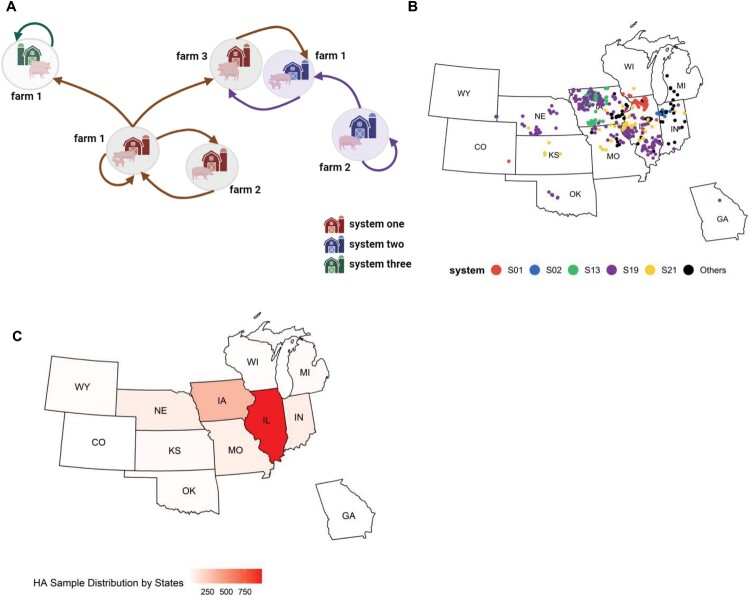
Illustration of data collection and sample distribution. The hierarchical structure of commercial farming operations encompasses systems, subsystems, and individual farms (A). A system consists of multiple subsystems and numerous farms, spanning intra – or inter-state levels, typically under the ownership of a single business entity. Subsystems generally denote a managerial or operational division. Swine farms are categorized based on the different stages of pig growth, including gestation, farrowing, nursery, rearing, and finishing phases. These farm types are further detailed in Figure 5. In this study, we curated 1909 IAV genomic sequences from 785 individual swine farms, representing 33 individual farming systems in the US (B). Sample collected farms belonging to different systems; colour coded by top 5 systems by sample count (C). Sample distribution by different states. Our dataset spans data collected from 12 states, primarily in the Midwest from 2004 to 2023. Among the states, Wyoming, Colorado, Michigan, and Georgia do not have sufficient data to meet our criteria for data preprocessing (see details in Materials and Methods) and thus were not included in the phylogeographic analyses (see Figures 4 and 5).

1909 IAV genomic sequences were obtained from archived diagnostic samples from twelve different states of the US, mainly from Illinois, between 2004 and 2023 (Figure 1) from the Disease Bioportal hosted at the Center for Animal Disease Modeling and Surveillance (CADMS) [29].

All IAV sequences were linked to associated metadata including information about the system, sub-system, farm site, farm type, and sample collection date. Addresses were geocoded using the Google Geocoding API, and verified manually using Google Earth imagery wherever necessary, yielding geographic coordinates for 1757 of 1909 sequences.

To ensure the validity of the genetic data analyses, we conducted a BLAST search with each gene sequence using data from the Influenza Research Database (IRD) [30]. Only segments matching an IAV segment were included in the subsequent analyses. For HA and NA genes, we assigned subtypes based on BLAST results. Four gene segments, HA (*n* = 1244 H1; *n* = 431 H3), NA (*n* = 68 N1; *n* = 103 N2), M (*n* = 58), and NS (*n* = 5), were identified.

In subsequent spatial analyses of IAV genetic lineage dispersal, we focused on HA sequences that meet the following criteria: (1) they include specific latitude and longitude information, and (2) the sequences come from farming sites with at least four samples to ensure robustness in the phylogeographic analyses. After applying these criteria, we had HA H1 sample data (*n* = 250) from 8 systems (S01, S21, S18, S02, S11, S19, S08, and S13) consisting of 45 individual farms from 8 states (Illinois, Missouri, Indiana, Wyoming, Nebraska, Kansas, Michigan, and Iowa) belonging to six different clades (1A.1.1, 1B.2.2.1, 1B.2.2.2, 1B.2.1, 1A.3.3.3, and 1A.3.3.2) for phylogeographic analysis. Similarly, HA H3 sample data (*n* = 67) consisted of 5 systems (S01, S21, S19, S08, and S02), 12 individual farming sites from 3 states (Illinois, Nebraska, and Missouri) with 2 clades (1990.4.a, 2010.1) obtained after applying the above filtering criterion to reduce the effect of data bias in phylogeographic analysis. Specifically, out of 250 H1, 176 sequences from 29 farms were identified and categorized as: Sow-farm (*n* = 40), Nursery (*n* = 4), Gilt-Development-Unit (*n* = 14), Nursery-to-Grower (*n* = 10), Grower-to-Finish (*n* = 30), Wean-to-Finish (*n* = 38), Finisher (*n* = 40); and the farm types for remaining 74 sequences were unknown. Of 67 H3 sequences, 54 belonged to 9 farms with different farm types as: Sow-farm (*n* = 9), Nursery (*n* = 10), Gilt-Development-Unit (*n* = 4), Grower-to-Finish (*n* = 4), Wean-to-Finish (*n* = 17), Finisher (*n* = 10); and the farm types for remaining 13 sequences were unknown.

### Data collection from public databases

To increase the genetic and geospatial coverage in our analyses, we downloaded swine-origin H1 (*n* = 8143) and H3 (*n* = 4402) IAVs from the Global Initiative on Sharing All Influenza Data (GISAID) [31], with collection dates from 2004 to 2023. To ensure computational feasibility and minimize potential biases in phylogeographic analyses, we randomly selected up to three sequences per H1 and H3 clade, matched for the year of collection and the state of origin. Consequently, a total of 68 H1 and 161 H3 public sequences were selected from public sequences. From these sequences, 36 H1 and 20 H3 were further matched based on the specific clades we collected from this study. In total, HA H1 (*n* = 286) and H3 (*n* = 87) including public and sample data were used in the subsequent phylogeographic analyses.

### Phylogenetic and phylogeographic analyses

Genetic clade classification was performed for H1 (*n* = 1244) and H3 (*n* = 431) viruses using a web-accessible subspecies classification tool which implements phylogeny-based global and US nomenclature system [8] with species filter as “Orthomyxoviridae – Swine Influenza H1,” “Orthomyxoviridae – Swine Influenza H3” respectively from the BV-BRC 3.33.1 [32].

Multiple sequence alignments were performed using MUSCLE v3.8.31 [33]. Then, we used MEGA-X [34], which implements a maximum-likelihood statistical approach to determine the best nucleotide substitution model for the sequence data. For each model, the parameters corrected Akaike information criteria (AIC), Bayesian information criterion (BIC), maximum-likelihood value, gamma (G), evolutionary variable (I), transition and transversion bias (R), nucleotide frequencies (f) and rate of substitution for nucleotide pairs (r) were calculated [34, 35]. The “General Time Reversible” model with gamma and invariant sites (GTR + G + I) performed the best as determined by BIC values and was selected in the following analyses.

Time-scaled Bayesian analyses with MCMC method were performed using BEAST v.1.10.4 [36], with the GTR + G + I substitution model with γ = 5 and a coalescent exponential growth prior to strict clock. The MCMC chain length was set at 100 million iterations with 20% burn-in and subsampling every 5000 or 10,000 iterations to improve the effective sample size (ESS) and enhance the convergence of the model. Tracer v1.7.2 [37] software was used to visualize and check if the model had converged or not using the prior value plot.

Bayesian Stochastic Search Variable Selection (BSSVS) method was used to determine the discrete phylogeographic diffusion of H1 or H3 genetic lineages across different farms within and across systems. A symmetric substitution model was applied, which performs discrete state ancestral reconstruction utilizing a standard continuous-time Markov chain (CTMC), ensuring that the migration rates between farming sites are reversible. Similarly, the virus migration rate among the different pig farms was calculated using the marginal approximation of the structured coalescent (MASCOT) [38] in BEAST.v2.7.6 [39]. Tracer v1.7.2 [37] was used to summarize the migration rates among different pig farms calculated using MASCOT. A higher migration rate between two farms suggests a more frequent exchange or higher connectivity between those farms.

SpreadD3 v0.9.7.1rc (2016) [40] software was used to analyse phylodynamic reconstructions derived from Bayesian analyses. The R package “ggtree” was used for tree visualizations [41]. Phylogeographic analysis was performed with sites having ≥4 samples to obtain ESS ≥200 to achieve a robust model performance and minimize the potential data bias.

Migration events were filtered for statistical significance using the criteria of Bayes factor ≥10 and posterior probability ≥0.7, for individual farming sites [42]. Different statistical support levels were defined as follows: 3≤ Bayes factor <10 indicates support; 10≤ Bayes factor <100 indicates strong support; 100 ≤ Bayes factor <1000 indicates very strong support; and Bayes factor ≥1000 indicates decisive support.

To address potential biases from uneven sampling, we applied two down-sampling strategies to assess the robustness of the migration events we identified: (1) reduced the number of samples from farms with a disproportionately large number of samples while retaining all farms in the analysis, and (2) randomly selected a portion of all samples, assuming the distribution reflects disease outbreak situations. The second approach may result in the loss of some samples and even some farms, particularly those with a small number of samples. In the first approach, we set a maximum of eight samples per farm and a minimum of four samples. For farms with more than eight samples, we randomly selected eight samples. In the second approach, we selected 80% of the samples and subjected them to the same model settings applied in our initial analysis. We repeated the analyses 10 times and generated bootstrap values for each migration event. The datasets from these down-sampling strategies were subjected to the phylogeographic analyses described earlier.

#### Statistical analyses

The non-parametric statistical test, Mann–Whitney U-test, was applied to compare the mean migration rates between two groups: within-system farms and across-systems farms. The null hypothesis (*H*_0_) states that there is no difference between the migration rates within and across the systems whereas the alternative hypothesis (*H*_1_) posits that the migration rates are different within and across the systems. If the *p*-value is less than 0.05, it indicates that there is a significance between the two groups, leading to the rejection of the null hypothesis.

#### Data availability

We have deposited the genomic data collected from this study in BioProject PRJNA1135970 with GenBank accession numbers PQ032940-PQ034436. This submission includes 1497 full lengths of HA sequences.

## Results

### Genetic diversity of the HA genes of swine IAVs

**Figure 2.**
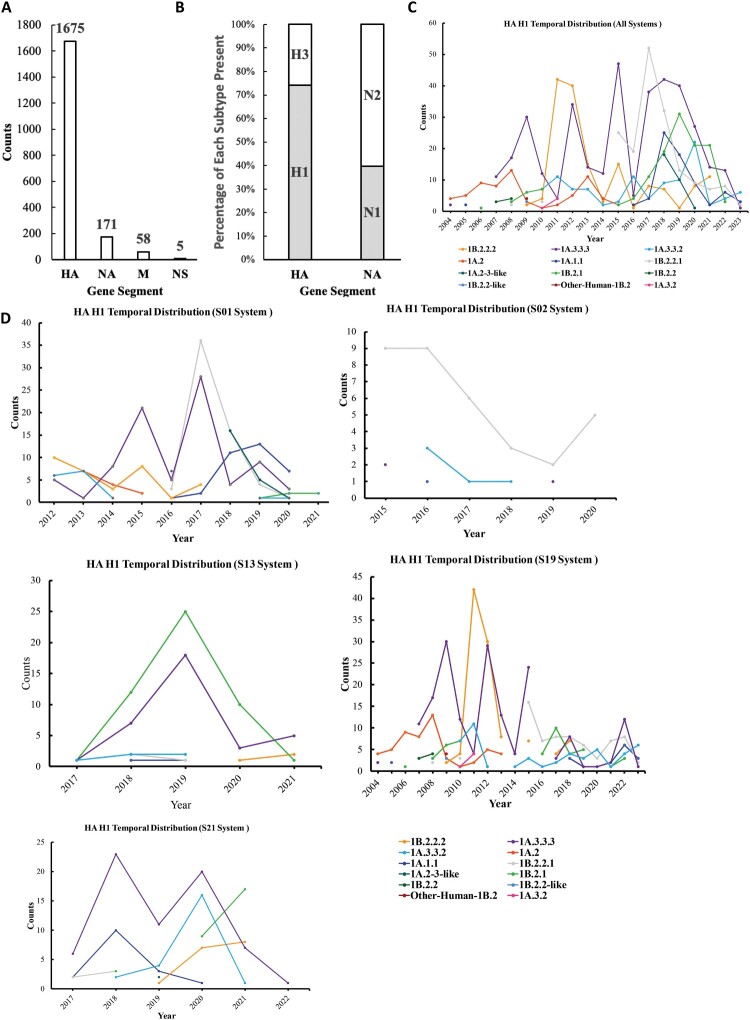
Genomic diversity of influenza data collected from this study. (A) Distribution of genome segments, including HA, M1, NA, and NS1, with HA being the most prevalent segment and NS1 the least frequent. (B) The distribution of HA and NA subtypes across the samples. (C) The temporal distribution of H1 clades/subclades from all systems. (D) The temporal distribution of H1 clades/subclades in the top five individual systems.

Sequence data were collected from 33 farming systems that collectively owned 785 pig farms spread across twelve states in the US: Colorado, Georgia, Illinois, Indiana, Iowa, Kansas, Kentucky, Michigan, Missouri, Nebraska, Wisconsin, and Wyoming, with most of the samples coming from Illinois (Figure 1(B)). The sequence data contained information for HA (1675 sequences), NA (171 sequences), M1 (58 sequences), and NS1 (5 sequences) gene segments (Figure 2(A)).

The HA sequences included 1244 (74.27%) H1 and 431 (25.73%) H3 genes (Figure 2(B)). The NA sequences included 68 (39.77%) N1 and 103 (60.23%) N2 genes (Figure 2(B)). Twelve genetic clades of H1 were detected, 1A.3.3.3, 1A.3.2, 1A.3.3.2, 1A.2, 1A.2.3-like, 1A.1.1, 1B.2.2.1, 1B.2.2.2, 1B.2.2-like, 1B.2.2, 1B.2.1, and other-human-1B.2. Among the H1 clades, 1A.3.3.3 was detected the most across the entire sampling period. Some clades, such as 1B.2.2.2, were found to persist for at least 12 years (Figure 2(C)). Similarly, for the H3 data, 13 genetic clades were detected, 1990.1, 1990.4, 2010.1, 2010.2, 1990.4.a, 1990.4.b1, 1990.4.b2, 1990.4.c, 1990.4.e, 1990.4.f, 1990.4.g, other-human-2010, and other-human-2010-like. Clades including 1990.4, 1990.4.a and 2010.1 were found to persist while other clades were only detected sporadically (Supplementary Fig. 1).

### Genetic diversity of H1 and H3 IAVs within and across swine farming systems

**Figure 3.**
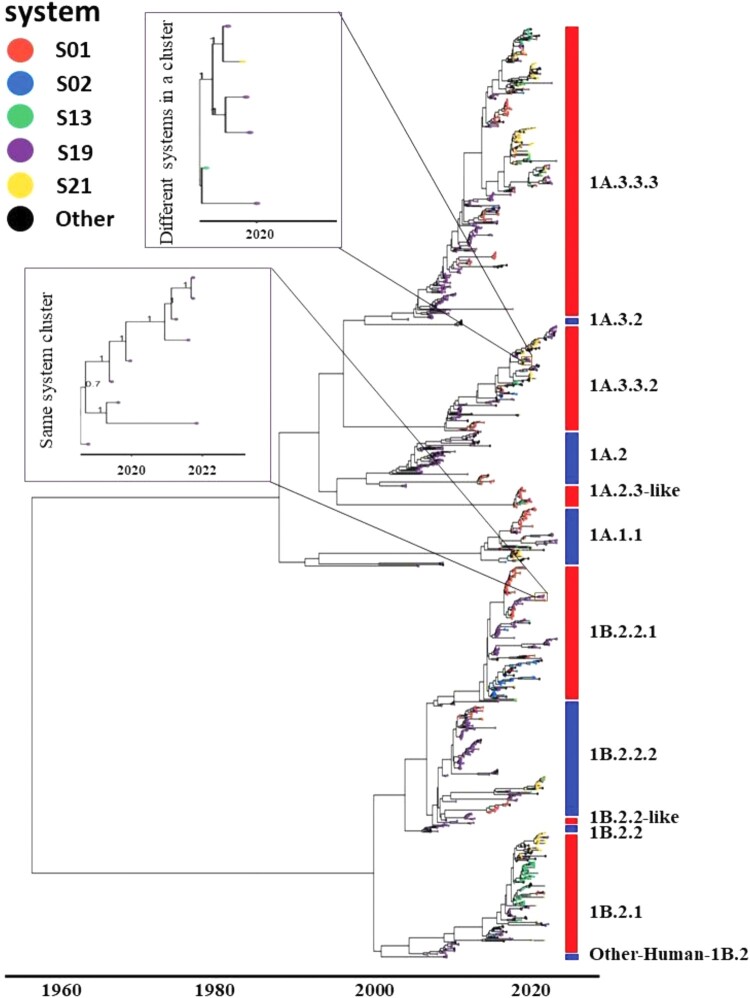
Phylogenetic tree of subtype H1 subtype swine IAVs in the US commercial swine farms (2004–2023). Colour coding of the tip labels was applied to represent the five most prevalent systems based on occurrence. H1 genes from other systems are labelled in black, while those lacking system information are marked in grey.

To understand the genetic diversity of the H1 viruses across different commercial swine systems, a Bayesian phylogenetic tree was generated for 1244 HA H1 nucleotide sequences, which represent IAVs sampled from 28 unique systems (Figure 3). A Maximum clade credibility tree showed the diversity by clade and depicted the genetic similarity of the circulating viruses at a given time among the systems. The top five systems (S01, S02, S13, S19, and S21) by occurrence of IAV sequences were colour coded to better visualize the high diversity within those systems. The results indicated that each of the 12 predominant genetic H1 clades/subclades was distributed across various systems, including the top five systems aforementioned (Figure 2(D)). Within each H1 clade/subclade, viruses from the same system tended to cluster together on the phylogenetic tree within specific time periods. For instance, the clade 1B.2.2.1 viruses were detected in system S19 from years 2018–2021 (Figure 3). However, it is noteworthy that the same genetic variant can co-circulate across different systems. For example, the 1A.3.3.2a viruses were detected across systems S13, S19 and S21 over the period of 2019–2020 (Figure 3).

Similarly, S01, S08, S13, S19 and S21 were the top five systems by the IAV sample occurrence for the HA H3 which were colour coded for ease of diversity visualization (Supplementary Fig. 1). As observed with HA H1, IAV samples from the same system typically tend to cluster together in HA H3 as well, such as system S01 and S19 in clade 1990.4. a.

### Dispersal of H1 and H3 genetic variants within and across swine farming systems

**Figure 4.**
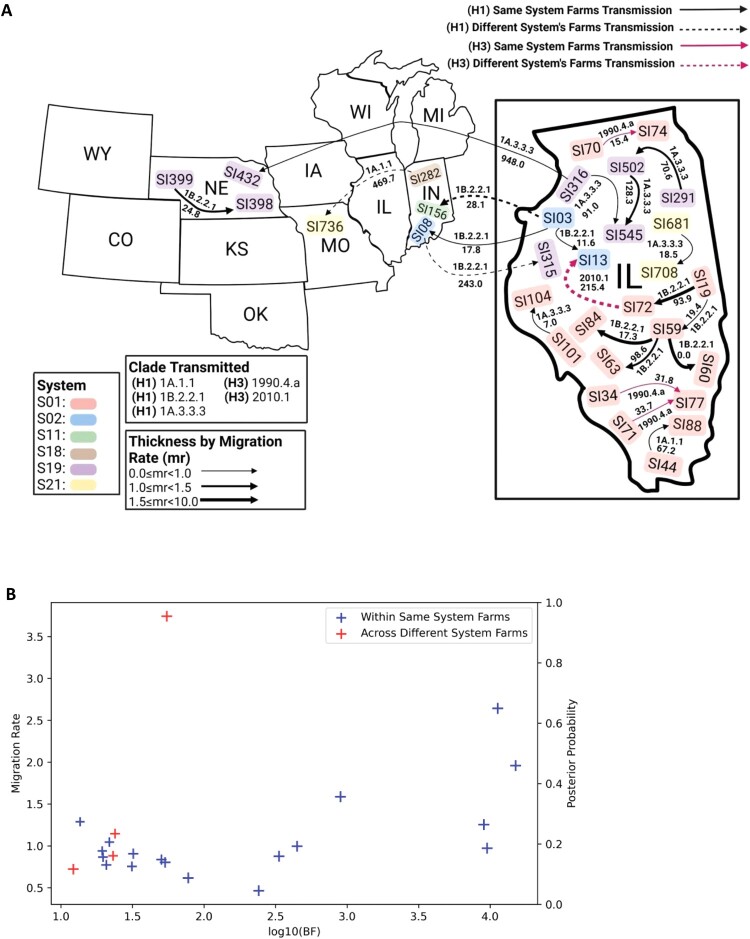
(A) Migration events of the H1 IAV clades 1A.1.1, 1B.2.2, and 1A.3.3.3 across different systems in US commercial swine farms. A Bayesian phylogeographic diffusion analysis was conducted using Bayesian stochastic search variable selection. A posterior probability ≥ 0.70 and a Bayes factor ≥ 10 were used as thresholds to robustly support the migration events among individual farm sites. Sites within the same system were grouped together and indicated by colour-coded boxes. The line thickness indicates the migration rate (mr) and the values along the lines are the clade and the distance between pig farms in kilometres (km) (B) Scatter plot for H1 and H3 showing the migration rate within and across systems’ farms supported by strong BF ≥10 and posterior probability ≥0.70.

To investigate the spatial dispersal patterns of the H1 and H3 genetic variants across the farms within and across swine farming systems, we conducted further phylogeographic analyses. For H1 virus, we focused on six genetic clades, including 1A.1.1, 1B.2.2.1, 1B.2.2.2, 1B.2.1, 1A.3.3.3 and 1A.3.3.2 (Figure 4(A)), that had been detected on at least two individual farms with at least ≥4 sequences to minimize the sampling bias and achieve ESS ≥200. Frequent migration events were detected to occur among farms belonging to the same system. For example, high frequency of intra-system transmission events occurred among farms SI19, SI44, SI59, SI60, SI63, SI72, SI84, SI88, SI101, and SI104 in system S01, and among farms SI291, SI316, SI502, and SI545 in system S19. However, the migration rates for the testing H1 genetic variants were not statistically significant within and across the systems farms (U-statistic  = 27.0, *p* = .65 > .05). Within each system, we found that certain farms play a more significant role in virus dispersal. For example, farm SI59 in system S01 was notably as a repeat source for intra-system virus dispersal, and farm SI03 in system S02 was highly involved in both inter – and intra-system dispersal events. Meanwhile, some farms, such as SI502 in system S19 and SI59 in system S01, were observed as both source and recipient of virus indicating a bi-directional dispersal pattern.

For HA H3 only two clades/subclades (1990.4.a, 2010.1) met the filtering criteria of having samples from at least two farms with a minimum of four samples per farm for the phylogeographic analysis. We observed a higher frequency of migration events among farms belonging to the same system, such as SI34, SI71, SI77, SI70 and SI74 within system S01. Additionally, inter-system farm migration was detected from SI72 in system S01 to SI13 in system S02. Similar to HA H1, the migration rates for the genetic variants of HA H3 were not statistically significant within and across the systems farms (U-statistic = 0, *p* = .50).

Our results also showed spatial transmission within and between systems, as were exemplified by similar viruses in clade 1A.1.1, 1B.2.2.1, and 1A.3.3.3 of H1 IAVs being detected in farms located in different states. For instance, directional transmissions were detected from Indiana to Missouri, from Illinois to Nebraska, from Illinois to Indiana, and from Indiana to Illinois at the farm level for both between and across systems. Bidirectional transmission of a clade 1B.2.2.1 of H1 IAV was observed between farms in Illinois and those in Indiana. System S02 was centrally involved in facilitating both inter – and intra-system transmission across these states. Among all identified transmission events in our datasets we collected, the majority were traced back to swine farms located in Illinois and Indiana.

To address potential biases from uneven sampling, we applied two down-sampling strategies to assess the robustness of the migration events we identified above (detailed in Materials and Methods). When we set a maximum of 8 samples per farm and a minimum of four samples, all migration events were consistently detected except for the event from SI03 to SI156 (Supplementary Table 1). During the bootstrapping analyses, among all detected migration events, only three were with a bootstrap value of 80% or more, including HA H1: SI03 to SI13, SI03 to SI156, and SI59 to SI84.

For HA H3, intra-system and inter-system farm spatial migration events were detected only in Illinois out of the three states (Illinois, Nebraska, and Missouri) participating in the analysis. Clade 1990.4.a was transmitted among farms within system S01, while clade 2010.1 clade was migrated from system S01 to system S02.

In summary, our findings showed that for HA H1 and H3 viruses, intra-system and inter-system transmission occurred between the swine farms, and intra-system transmissions were relatively more frequent, but the migration rate was not significantly different (U-statistic  = 31.0, *p* = .71 for H1 and H3 combined). Additionally, transmissions can be bidirectional between farms and states.

### Dispersal of H1 and H3 genetic variants through swine farming system compartments

**Figure 5.**
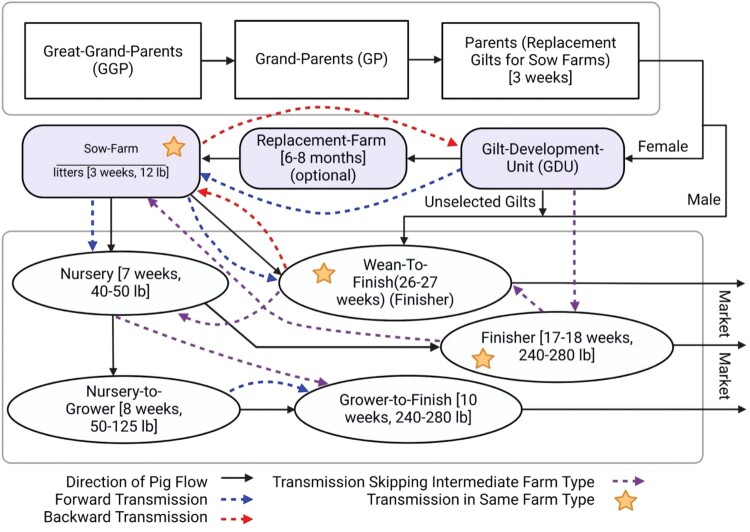
Distribution of H1 IAV migration events within a typical commercial swine operation infrastructure. The operation begins with Great-Grand-Parents (GGP), genetically selected pigs that produce Grand-Parents (GP), who in turn breed parent pigs destined to replenish the population in Sow-Farms (SF). Male pigs are usually sent directly from weaning to Wean-to-Finish (WF) farms, while females are transferred to Gilt-Development-Units (GDU). The most suitable gilts from GDUs are then moved to SF, although some systems may place them in a replacement farm before their final transfer to SF. The farming practices vary across different systems, but the typical pathways post-farrowing at SF include: (i) from Nursery to Finisher farm, (ii) from Nursery to Nursery-to-Grower and then to Grower-to-Finish farm, and (iii) directly from SF to Wean-to-Finish (WF) farm.

In intense swine farming operations, pigs are regularly transported between different types of farms, at various stages of their growth (Figure 5). Pig production typically begins at the Great-Grand-Parents (GGP) level, the breeding pyramid, which produces Grand-Parents animals (GPs). These GPs are then bred to produce parent gilts, intended as replacements for Sow-Farms. From the offspring of these parents, only female pigs are selected for transfer to Gilt-Development-Units (GDU). In the GDU, these chosen gilts spend six to eight months before being moved to Replacement-Farms, and eventually to Sow-Farms. Depending on the specific setup of the system, pigs are transported from Sow-Farms to various types of farms, such as Nursery or Wean-to-Finish, with the latter being the most common pathway in recent days. In some systems, pigs may move from a Nursery to a Finisher directly, or they may go from Nursery to a Nursery-to-Grower and then to a Grower-to-Finish. The logistical intricacies of moving pigs between farms at specific life stages are crucial in the pig farming industry. Any interruptions or delays in this process can disrupt the entire production cycle, leading to significant economic losses. This constant need for pig replacement exerts pressure on farm operations, necessitating the transport of pigs to various locations sometimes to nearby farms within the same state, and other times to distant farms in another state or even to farms within a different production system.

This transportation of pigs across different locations and systems inherently increases the risk of influenza transmission. To explore the factors contributing to the spatial dispersal dynamics of swine IAVs, we conducted an analysis of the relationship between migration events and the various compartments of the swine farming system. As a result, we observed IAV migration events across different swine farm types. Notably, GDUs within the S19 system facilitated the dispersal of the 1A.3.3.3 clade of H1 IAV from GDU farm SI316 to the Finisher farm SI432 and the Sow-Farm SI545, all within the same system where the farms SI316 and SI545 are located in Illinois, while SI432 is in Nebraska. Similarly, the dispersal of HA H3 clade 1990.4.a was detected from Finisher farm SI34 to Wean-to-Finish SI77 and from Wean-to-Finish SI70 to Nursery SI74, all located in Illinois and belonging to system S01. Furthermore, we observed dispersal of the genetic lineages between the same type of farms (e.g. from one Sow-Farm to another Sow-Farm and from one Finisher to another Finisher) belonging to the same system but at different locations within the same state. For example, the 1B.2.2.1 clade of H1 IAV was transmitted between the Sow-Farms SI19 and SI59 within system S01, and the 1A.3.3.3 clade of H1 IAV between the Finisher farms SI291 and SI502 within the S19 system where all farms were located in Illinois.

In the S01 system, most dispersal events occurred in line with the direction of pig replacement across different life stages, such as from Sow-Farms to Nursery and from Sow-Farms to Wean-to-Finish. However, genetic dispersal in the opposite direction of pig flow across specialized farm types i.e. from Sow-Farms to GDUs and from Wean-to-Finish back to Sow-Farms was also observed in system S01. Migration within the same farm type Wean-to-Finish, was also observed in system S01. Similarly, within the S02 system, intra-system migration of the 1B.2.2.1 clade of H1 IAV occurred both within and across state lines, affecting different farm types, such as the migration from Nursery-to-Grower farm SI03 in Illinois to Grow-to-Finish farm SI08 where farms SI08 and SI13 are from Indiana and Illinois respectively.

In summary, our analyses identified frequent movements of IAVs between swine farm types within and across farming systems. IAV transmission also occurred primarily, but not exclusively, along with the expected patterns of pig routine replacement.

## Discussion

Since the mid-1990s, multiple genetic variants of H1 and H3 IAVs have been co-circulating in US commercial swine farms [5–7]. These genetically diverse H1 and H3 viruses are antigenically different, showing different extents of cross-reactivity in serologic assays [43, 44]. Our genetic analysis of over 1900 curated HA sequences within 12 H1 clades/subclades and 13 H3 clades/subclades over the course of approximately 20 years highlights the genetic diversity of IAVs enzootic in the US swine population (Figure 2(C), Supplementary Fig. 1). The genetic clades we identified matched all clades/subclades from public databases during a similar timeframe and within the same region, as illustrated in Figure 3. We identified co-circulation of both H1 and H3 viruses with multiple clades/subclades present within the same farming system. However, the diversity of viruses observed across different systems exceeded that found within any single system (Figure 2(D)).

We found that H1 and H3 virus clades across different types of farms within the same farming system often grouped together, suggesting active virus movement across phase of a swine production system, which was further supported by Bayesian phylogeographic analyses (Table 1; Table 2). Persistence of similar viruses and introduction of new lineage through transmission in intense commercial farms could promote the emergence of novel genetic variants through rapid mutations or genetic reassortment. In addition, previous research has demonstrated that antigenic drift, caused by mutations at antigenic sites, enables IAV to reinfect swine, evading prior immunity [45, 46]. This could particularly be important in Sow-Farms, which are often vaccinated with influenza vaccines and are more prone to multiple IAV exposures due to their longer life span. Our analysis revealed that Sow-Farms are among the primary sources of transmitted virus which has also been reported in other studies in the US [28, 47] (Figure 4). H1 viruses from different systems can occasionally be found within a specific genetic cluster, supportive of virus dispersal between systems, a finding supported by Bayesian phylogeographic analyses (Table 1). Such inter-system transmission not only enables the retention of existing viruses but also facilitates the emergence of new strains through the reassortment of viruses from two different farming systems. Overall, these findings imply that the dispersal of IAV genetic variants across different farms and systems might be a crucial mechanism supporting virus persistence in commercial farming environments. Typically, dispersal occurs during routine swine movements among the farms [24], when infected hogs come in direct contact with others [48, 49] and other factors like infected human-pig interaction in farms, reuse of infected items, aerosols and air exhaustion also play important role in IAV transmission [50].

**Table 1.** List of genetic migration events of H1 IAVs detected by Bayesian analyses.

Donor				Recipient				Distance (km)	Clade	Bayes factor	Posterior probability
Farm	Farm type	System	State	Farm	Farm type	System	State				
SI44	Wean-to-Finish	S01	Illinois	SI88	Sow-Farm	S01	Illinois	67.17	1A.1.1	77.46	0.99
SI19	Sow-Farm	S01	Illinois	SI59	Sow-Farm	S01	Illinois	19.41	1B.2.2.1	19.65	0.78
SI19	Sow-Farm	S01	Illinois	SI72	Nursery	S01	Illinois	93.90	1B.2.2.1	15051.33	1.00
SI59	Sow-Farm	S01	Illinois	SI84	Wean-to-Finish	S01	Illinois	17.27	1B.2.2.1	897.77	0.99
SI59	Sow-Farm	S01	Illinois	SI63	Wean-to-Finish	S01	Illinois	98.56	1B.2.2.1	13.60	0.71
SI59	Sow-Farm	S01	Illinois	SI60	Gilt-Development-Unit	S01	Illinois	0.00	1B.2.2.1	11287.09	1.00
SI101	Finisher	S01	Illinois	SI104	Wean-to-Finish	S01	Illinois	6.97	1A.3.3.3	9523.45	1.00
SI03	Nursery-to-Grower	S02	Illinois	SI08	Grow-to-Finish	S02	Indiana	17.77	1B.2.2.1	333.99	0.98
SI03	Nursery-to-Grower	S02	Illinois	SI13	Grow-to-Finish	S02	Illinois	11.56	1B.2.2.1	241.19	0.98
SI03	Nursery-to-Grower	S02	Illinois	SI156	Grow-to-Finish	S11	Indiana	28.11	1B.2.2.1	23.86	0.81
SI08	Nursery-to-Grower	S02	Indiana	SI315	Unknown	S19	Illinois	243.04	1B.2.2.1	23.11	0.80
SI282	Unknown	S18	Indiana	SI736	Unknown	S21	Missouri	469.66	1A.1.1	12.19	0.95
SI399	Unknown	S19	Nebraska	SI398	Unknown	S19	Nebraska	24.08	1B.2.2.1	9028.54	1.00
SI291	Finisher	S19	Illinois	SI502	Finisher	S19	Illinois	70.60	1A.3.3.3	446.69	0.98
SI316	Gilt-Development-Unit	S19	Illinois	SI432	Finisher	S19	Nebraska	984.03	1A.3.3.3	19.43	0.73
SI316	Gilt-Development-Unit	S19	Illinois	SI545	Sow-Farm	S19	Illinois	91.01	1A.3.3.3	50.32	0.88
SI502	Finisher	S19	Illinois	SI545	Sow-Farm	S19	Illinois	128.33	1A.3.3.3	21.78	0.75
SI681	Unknown	S21	Illinois	SI708	Unknown	S21	Illinois	18.50	1A.3.3.3	31.31	0.81

**Table 2.** List of genetic migration events of H3 IAV detected by Bayesian analyses.

Donor				Recipient				Distance (km)	Clade	Bayes factor	Posterior probability
Farm	Farm type	System	state	Farm	Farm type	System	state				
SI34	Finisher	S01	Illinois	SI77	Wean-to-Finish	S01	Illinois	31.83	1990.4.a	53.44	0.94
SI70	Wean-to-Finish	S01	Illinois	SI74	Nursery	S01	Illinois	15.38	1990.4.a	31.96	0.90
SI71	Wean-to-Finish	S01	Illinois	SI77	Wean-to-Finish	S01	Illinois	33.67	1990.4.a	20.75	0.85
SI72	Nursery	S01	Illinois	SI13	Grow-to-Finish	S02	Illinois	215.41	2010.1	54.90	0.99

Our analyses uncovered some interesting patterns of IAV transmission. For instance, the spread of virus was not confined to the state level; it sometimes extended to distant sites in another state, either within the same system or across different systems in the Midwest. This highlights an additional layer of complexity in virus transmission at regional levels, supplementing previous findings that documented virus circulation within the same state [28] or across broader areas of the US, including the Midwest and Southeast regions [27]. In addition, we did detect virus dispersal between farms in both directions of swine routine replacement flow, though dispersal typically followed pig movement. For instance, bidirectional dispersal between different types of farms, such as from Sow-Farms to Wean-to-Finish and *vice versa*, was observed. Additionally, there were occasions where virus dispersal occurred directly between non-adjacent stages in the production chain, for example, from GDU directly to Finisher, bypassing intermediate farming types. Most of the observed transmission occurred between the farms separated by <100 Km but some spread across much larger distance e.g. the virus dispersal between farms SI316 and SI432, between farms SI08 and SI315, and between farms SI282 and SI736 where the distance between them is 984.03, 243.04 and 469.66 km respectively. Similar patterns were observed in H3 genetic variants, where migration events happened more frequently: (1) intra-system than inter-system, mostly within the same state at distances ≤100 km, (2) among farm types such as Wean-to-Finisher, (3) between farms without intermediate farm types, such as Finisher to Wean-to-Finish, and (4) in the reverse direction, such as Wean-to-Finisher to Nursery (Table 2; Figure 4(A)). Overall, our findings of inter- and intra-system transmission events occurring among farms can be located in either close or distant geographical proximity.

Previous studies suggested that, in addition to pig movement, other factors such as swine handling [51, 52], fomites [53], and aerosols [54, 55], infected swine workers [56], and unsafe handling and reuse of the infected materials [57], may contribute to the aforementioned transmission events. Indeed, over the past decade, the US swine industry has implemented a series of practices to mitigate various IAV transmission risks. These include restricting worker access to multiple farms, implementing shower-in and shower-out protocols, installing air filters, providing special care for swine workers [56, 58], and disinfecting items reused during swine handling. Nevertheless, the occurrence of IAV transmission across farms still does not appear to be uncommon, suggesting that more detailed epidemiological studies are necessary to pinpoint the major factors influencing transmission, likely varying by system or farm.

The limitations of this study include the use of an opportunistic dataset, which influenced the selection of sequences, farms, and systems included in this analysis, along with the relatively small sample sizes employed. Consequently, the samples utilized may not comprehensively represent the entire genomic diversity and their distribution across the studied farms. Since our analysis of virus transmission relies on genomic sequences, it was limited to samples with high RNA quality necessary for obtaining high-quality genomic sequences. Consequently, those transmission events associated with low-quality RNA or asymptomatic cases are likely to be missed. Thus, it is probable that our analyses overlooked transmission events among the farms covered in our study. For example, the failure to detect transmission events across farms at non-adjacent stages of the production chain could potentially be attributed to the limited size of the samples. The potential missing transmission events were highlighted by the results from our down-sampling analyses. Specifically, the majority of transition events remained consistently confident when we reduced sample sizes for specific farms while keeping those farms in the analysis. However, the confidence in transition events decreased when some samples were randomly removed, leading to the exclusion of certain farms from the analyses. Thus, further studies are necessary to implement a systematic sampling strategy, aiming to achieve a high-resolution understanding of the transmission patterns at each stage of the swine life cycle.

In summary, the extensive analysis we performed in this study at both the system and farm level highlights the complex dynamics of IAV transmission across various compartments and locations within the swine farming infrastructure. We observed significant intra-system genetic dispersal of IAVs among farms situated in the same region, while the spread of the virus to geographically distant farms, both within and between systems, was comparatively rare. These findings demonstrate that the transmission of IAV is affected not just by the movement of pigs but also by additional factors, underscoring the crucial role of farm management practices in preventing and controlling swine influenza. More frequent migration of IAV within systems than between systems indicates that prioritizing resource allocation to control and mitigate the viral infection and persistent at the system level, particularly in systems consistently facing influenza challenges, could be effective. Focusing on key source farms, like Sow-Farms, may represent an efficient strategy for managing influenza in commercial swine operations in the United States.

## Supplementary Material

SupFig1.tif

Supplementary Information.docx
