# Supplementary material for: Spatial patterns of influenza A virus spread across compartments in commercial swine farms in the United States
Source: Emerg Microbes Infect. 2024 Sep 2;13(1):2400530. doi: 10.1080/22221751.2024.2400530 (PMC11445930; doi:10.1080/22221751.2024.2400530)
Supplement: Supplementary Information.docx [file TEMI_A_2400530_SM5128.docx]

**Supplementary Information**

**Supplementary Figure 1.** Genomic diversity of H3 subtype swine IAVs in the US commercial swine farms (2004 to 2023). **A)** Temporal dynamics of H3 clades/subclades; **B)** Phylogenetic tree of subtype H3 subtype swine IAVs.

**Supplementary Table 1.** List of genetic migration events of H1 IAVs detected by Bayesian analyses (down scale analysis).

| **Donor** | | | | **Recipient** | | | | **Distance(km)** | **Clade** | **Bayes Factor** | **Posterior Probability** |
| --- | --- | --- | --- | --- | --- | --- | --- | --- | --- | --- | --- |
| **Farm** | **Farm Type** | **System** | **State** | **Farm** | **Farm Type** | **System** | **State** |  |  |  |  |
| SI44 | Wean-to-Finish | S01 | Illinois | SI88 | Sow-Farm | S01 | Illinois | 67.17 | 1A.1.1 | 69.77 | 1.00 |
| SI19 | Sow-Farm | S01 | Illinois | SI59 | Sow-Farm | S01 | Illinois | 19.41 | 1B.2.2.1 | 19.57 | 0.78 |
| SI19 | Sow-Farm | S01 | Illinois | SI72 | Nursery | S01 | Illinois | 93.90 | 1B.2.2.1 | 45729.85 | 1.00 |
| SI59 | Sow-Farm | S01 | Illinois | SI84 | Wean-to-Finish | S01 | Illinois | 17.27 | 1B.2.2.1 | 573.28 | 0.99 |
| SI59 | Sow-Farm | S01 | Illinois | SI63 | Wean-to-Finish | S01 | Illinois | 98.56 | 1B.2.2.1 | 14.01 | 0.71 |
| SI59 | Sow-Farm | S01 | Illinois | SI60 | Gilt-Development-Unit | S01 | Illinois | 0.00 | 1B.2.2.1 | 15239.52 | 1.00 |
| SI101 | Finisher | S01 | Illinois | SI104 | Wean-to-Finish | S01 | Illinois | 6.97 | 1A.3.3.3 | 57176.45 | 1.00 |
| SI03 | Nursery-to-Grower | S02 | Illinois | SI08 | Grow-to-Finish | S02 | Indiana | 17.77 | 1B.2.2.1 | 381.94 | 0.99 |
| SI03 | Nursery-to-Grower | S02 | Illinois | SI13 | Grow-to-Finish | S02 | Illinois | 11.56 | 1B.2.2.1 | 150.45 | 0.96 |
| SI08 | Nursery-to-Grower | S02 | Indiana | SI315 | Unknown | S19 | Illinois | 243.04 | 1B.2.2.1 | 18.86 | 0.77 |
| SI282 | Unknown | S18 | Indiana | SI736 | Unknown | S21 | Missouri | 469.66 | 1A.1.1 | 12.77 | 0.95 |
| SI399 | Unknown | S19 | Nebraska | SI398 | Unknown | S19 | Nebraska | 24.08 | 1B.2.2.1 | 4567.90 | 1.00 |
| SI291 | Finisher | S19 | Illinois | SI502 | Finisher | S19 | Illinois | 70.60 | 1A.3.3.3 | 352.50 | 0.98 |
| SI316 | Gilt-Development-Unit | S19 | Illinois | SI432 | Finisher | S19 | Nebraska | 984.03 | 1A.3.3.3 | 17.92 | 0.71 |
| SI316 | Gilt-Development-Unit | S19 | Illinois | SI545 | Sow-Farm | S19 | Illinois | 91.01 | 1A.3.3.3 | 41.90 | 0.85 |
| SI502 | Finisher | S19 | Illinois | SI545 | Sow-Farm | S19 | Illinois | 128.33 | 1A.3.3.3 | 21.46 | 0.75 |
| SI681 | Unknown | S21 | Illinois | SI708 | Unknown | S21 | Illinois | 18.50 | 1A.3.3.3 | 35.53 | 0.83 |
